# Associations Between Sleep and Non-Motor Symptoms in RBD-Screened Suspected Prodromal Parkinson’s Disease with Hyposmia or Orthostatic Hypotension

**DOI:** 10.3390/jcm15145371

**Published:** 2026-07-09

**Authors:** Erlandas Paulėkas, Rugilė Mučaitė, Evelina Pajėdienė, Abdonas Tamošiūnas, Vaiva Lesauskaitė, Kęstutis Petrikonis

**Affiliations:** 1 Department of Neurology, Lithuanian University of Health Sciences, LT-44307 Kaunas, Lithuaniakestutis.petrikonis@lsmu.lt (K.P.); 2 Institute of Cardiology, Lithuanian University of Health Sciences, LT-44307 Kaunas, Lithuania

**Keywords:** Parkinson’s disease, suspected prodromal Parkinson disease, non-motor symptoms, REM sleep behavior disorder, excessive daytime sleepiness, insomnia, hyposmia

## Abstract

**Background and Objectives:** Parkinson’s disease (PD) includes a prodromal phase characterized by non-motor symptoms (NMS). We investigated whether sleep disturbances co-aggregate with other NMS in suspected prodromal PD (p-PD) to refine early phenotyping and risk stratification. **Materials and Methods:** A population-based survey was conducted in Kaunas, Lithuania. Participants from the Kaunas City preventive health screening cohort (2024–2025) were screened using the Innsbruck REM Sleep Behaviour Disorder Inventory (RBD-I). Sixty-two individuals were included: 31 with suspected p-PD and 31 controls. Demographic characteristics, clinical features, sleep symptoms, olfactory function, autonomic symptoms, cognition, and mood were assessed. Between-group comparisons and within-group correlation analyses were performed. **Results:** Groups did not differ in age, sex, education, lifestyle exposures, or family history (all *p* > 0.05). Compared with controls, individuals with suspected p-PD had a higher prevalence of insomnia (71.0% vs. 12.9%, *p* < 0.001), pathological daytime sleepiness (22.6% vs. 0%, *p* = 0.005), cognitive impairment (54.8% vs. 16.1%, *p* = 0.001), and constipation (32.3% vs. 0%, *p* = 0.002). As expected from the predefined enrichment strategy, hyposmia and orthostatic hypotension (OH) were observed only among suspected p-PD participants. Within the suspected p-PD group, the Non-Motor Symptoms Questionnaire (NMSQ) scores correlated with depressive/anxiety symptoms (r = 0.578, *p* = 0.001). Olfactory dysfunction correlated with cognitive impairment (r = 0.410, *p* = 0.001) and depressive/anxiety symptoms (r = 0.396, *p* = 0.001). **Conclusions:** Sleep disturbances, particularly insomnia and excessive daytime sleepiness, co-occurred with cognitive, affective, and gastrointestinal non-motor manifestations in individuals with suspected p-PD, supporting the concept of a broader prodromal phenotype. Comprehensive screening in individuals with RBD symptoms may improve early PD risk stratification beyond single-marker approaches.

## 1. Introduction

Parkinson’s disease (PD) is a progressive neurodegenerative disorder characterized by a wide range of motor and non-motor symptoms (NMS) [1]. NMS include sleep disturbance, hyposmia, autonomic dysfunction, cognitive change, and altered mood/affect. These symptoms can substantially affect quality of life and often precede motor onset by years to decades [2]. A probabilistic prodromal stage has been formalized using multimodal markers, most particularly in the Movement Disorder Society (MDS) research criteria for probable prodromal PD (p-PD) [3]. Among these markers, REM sleep behavior disorder (RBD), hyposmia, and orthostatic hypotension (OH), especially in combination, are among the strongest clinical indicators of phenoconversion to α-synucleinopathy [4,5]. Early involvement of brainstem, olfactory, and limbic circuits with widespread α-synuclein pathology provides a biological rationale for these prodromal features [1,6]. Several studies have highlighted the importance of sleep disturbances during the prodromal and early stages of Parkinson’s disease. Sleep abnormalities frequently coexist with other non-motor manifestations, including depressive symptoms, cognitive changes, autonomic dysfunction, and olfactory impairment [7,8]. Emerging evidence suggests that these NMS tend to cluster rather than occur in isolation, supporting the concept of distinct prodromal phenotypes characterized by overlapping sleep, affective, cognitive, and autonomic features. These observations indicate that sleep disturbances may represent an important component of broader prodromal phenotypes and may improve risk stratification when considered alongside other established prodromal markers [8,9]. However, most previous studies have focused on isolated prodromal markers or on established Parkinson’s disease, whereas the relationships between common sleep complaints such as insomnia and excessive daytime sleepiness (EDS) and other non-motor manifestations in individuals with suspected p-PD remain incompletely understood. In particular, data are limited regarding how sleep disturbances co-aggregate with olfactory dysfunction, autonomic abnormalities, mood symptoms, and cognitive impairment within enriched prodromal cohorts [10,11]. Therefore, we investigated whether sleep disturbances (insomnia, EDS) were associated with other NMS in suspected p-PD individuals defined by RBD symptoms plus hyposmia or OH.

## 2. Materials and Methods

### 2.1. Study Population

**Figure 1:**
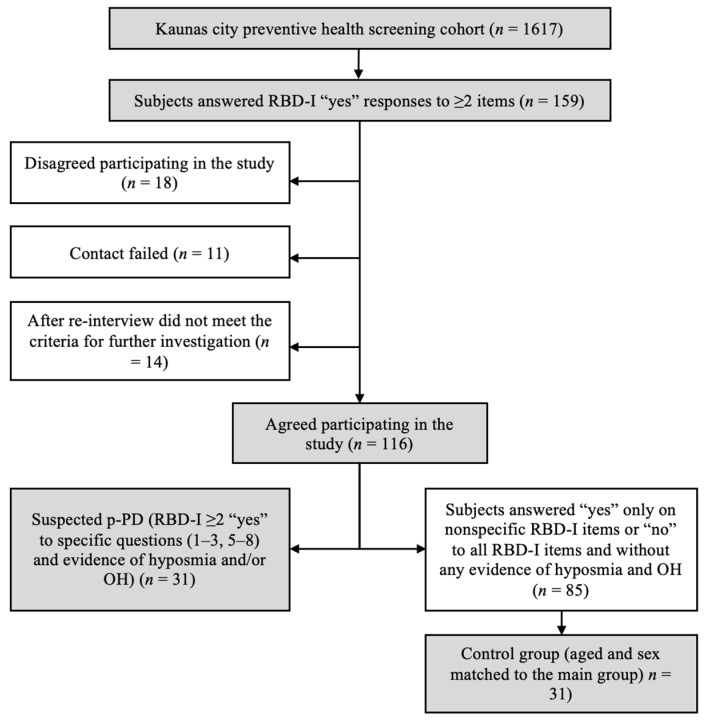
Flow diagram of group assignment and assessments. Abbreviations: RBD-I—REM Sleep Behavior Disorder Inventory; p-PD—Prodromal Parkinson’s disease; OH—orthostatic hypotension.

We conducted a cross-sectional, case–control study within the Kaunas city preventive health screening cohort (*n* = 1617). The baseline cohort screening survey was conducted in 2024–2025. Participants eligible for the present study were subsequently invited for additional in-person assessments performed in 2025. The in-person evaluations were conducted between April and June 2025. From the screening cohort (*n* = 1617), individuals who answered “yes” to ≥2 items on the Innsbruck REM Sleep Behavior Disorder Inventory (RBD-I) were identified (*n* = 159) and invited for in-person evaluation. Of these, 116 agreed to participate after re-contact. 18 individuals declined participation, 11 could not be contacted, and 14 did not meet eligibility criteria at re-interview. All 116 participants underwent the study assessments. Based on the predefined classification criteria, 31 participants were assigned to the suspected p-PD group. The remaining 85 participants constituted the pool of potential controls because they reported “yes” responses only to non-specific RBD-I items and had no evidence of hyposmia or OH. To obtain comparable groups, 31 controls were selected from this pool using age- and sex-matching at a 1:1 ratio with the suspected p-PD group. Consequently, the final analytical sample consisted of 62 participants: 31 individuals with suspected p-PD and 31 matched controls (Figure 1). The present study represents a secondary analysis of participants derived from the larger Kaunas city preventive health screening cohort. No formal a priori sample size calculation was performed specifically for this analysis; the final sample size was determined by the number of participants meeting the predefined criteria for suspected p-PD and completing all study assessments. The study complied with the Declaration of Helsinki and was approved by the LUHS Bioethics Committee (protocol code 2025-BEC2-0305).

### 2.2. Group Assignment and Assessment of Suspected Prodromal Features

Inclusion criteria were age ≥18 years, participation in the 2024–2025 preventive health screening program, and RBD-I “yes” responses to ≥2 items. Exclusion criteria were new diagnosis of a neurological disease, structural brain injury, or other somatic illness likely to substantially disturb sleep; inability or unwillingness to provide informed consent; or logistical/technical barriers precluding participation.

RBD-I item responses were used for initial screening. Video polysomnography (v-PSG) was not performed; therefore, RBD was considered suspected rather than confirmed. Following the comprehensive clinical evaluation (see below) and consistent with the validated RBD-I structure, questionnaire items were classified as “specific” (items 1–3 and 5–8) or “non-specific” (items 4 and 9) [12]. Participants were then classified into one of the following groups:

- Suspected p-PD group: RBD-I ≥ 2 “yes” on specific items and evidence of hyposmia and/or OH, chosen a priori as prodromal markers with among the highest positive likelihood ratios in the updated MDS Research Criteria for p-PD [3]. The combination of RBD symptoms with hyposmia and/or OH was used to enrich the cohort for individuals with a higher estimated probability of suspected p-PD.
- Control group: “yes” only on nonspecific RBD-I items, without evidence of hyposmia or OH (Figure 1).

Demographic and clinical variables. Standardized case report forms captured sex, age, education, marital status, occupation, work with metals or in polluted environments, childhood drinking water source, physical activity habits, dominant hand, and family history of PD, current medication use and comorbidities.

Autonomic function. Active orthostatic testing was performed after 5 min supine rest by measuring blood pressure (BP) and heart rate (HR) supine and at 1 and 3 min after active standing. OH was defined as a sustained decrease in systolic BP (SBP) ≥ 20 mmHg or diastolic BP (DBP) ≥ 10 mmHg within 3 min of standing; a blunted chronotropic response (ΔHR/ΔSBP < 0.5 bpm/mmHg) suggested neurogenic OH. Stool consistency was assessed using the Bristol Stool Form Scale.

Non-motor symptoms. Global NMS burden was assessed with the NMSQ. Olfactory function was evaluated using the 12-item smell identification test (SS-12); scores were categorized as normosmia (9–12), hyposmia (5–8), or anosmia (0–4). Cognition was assessed with the Montreal Cognitive Assessment (MoCA; 0–30). For descriptive purposes, scores were grouped as mild (18–25), moderate (10–17), or severe (<10) impairment. Anxiety and depression were measured with the Hospital Anxiety and Depression Scale (HADS; Anxiety 0–21, Depression 0–21), categorized as normal (0–7), borderline (8–10), or clinically significant (≥11).

Sleep-related instruments. All participants completed Lithuanian-language versions of the Insomnia Severity Index (ISI), Berlin Sleep Apnea Questionnaire (BQ), Restless Legs Syndrome Rating Scale (IRLS), ESS, RBD-I. Previously published Lithuanian translation, cross-cultural adaptation, and/or psychometric validation studies are available for the ISI, IRLS, RBD-I [13,14,15]. The ESS and BQ were administered using Lithuanian-language versions routinely employed in the sleep laboratory.

Specific sleep-related questionnaire evaluation thresholds are presented in Table 1.

### 2.3. Statistical Analysis

Analyses were conducted in IBM SPSS Statistics v30.0.0.0 (IBM Corp., Armonk, NY, USA). Descriptive statistics for continuous variables included mean, standard deviation, and range, whereas categorical variables were summarized as counts and percentages. Between-group comparisons for non-normally distributed continuous data were performed using the Mann–Whitney U test. Associations within the suspected p-PD group were examined using Spearman’s rank correlation. Categorical variables were compared using the chi-square test or Fisher’s exact test when expected cell counts were small. All tests were two-tailed with a significance level of α = 0.05.

The correlation analyses were exploratory and intended to identify potential associations between sleep-related measures and non-motor features in suspected p-PD. Given the modest sample size and hypothesis-generating nature of these analyses, no formal correction for multiple comparisons was applied; therefore, the reported *p*-values should be interpreted as exploratory.

## 3. Results

Demographic and clinical characteristics of participants with suspected p-PD were broadly comparable to controls. Detailed descriptive statistics are provided in Table 2.

### 3.1. Sleep Disturbances in Participants with Suspected p-PD and Controls

Between-group comparison revealed several differences. The mean ISI score was significantly higher in the suspected p-PD group than in controls (11.19 ± 6.33 vs. 4.52 ± 3.40, *p* < 0.001). Consistent with this finding, moderate (25.8%) and severe (3.2%) insomnia occurred only in the suspected p-PD group, whereas most controls had no insomnia (87.1%) or only subthreshold symptoms (12.9%). On the Berlin Sleep Apnea Questionnaire (BQ), the proportion at high risk for obstructive sleep apnea (OSA) was higher in the suspected p-PD group (61.3% vs. 41.9%) but not statistically significant (|Z| = 1.512, *p* = 0.130). Restless Legs Syndrome ratings (IRLS) did not differ significantly (|Z| = 0.495, *p* = 0.621), although mild–moderate RLS was more frequent in the suspected p-PD group and absent in controls. The Epworth Sleepiness Scale (ESS) score was significantly higher in the suspected p-PD group (7.23 ± 4.28 vs. 5.90 ± 5.10; |Z| = 2.786, *p* = 0.005). Pathological daytime sleepiness was observed in 22.6% of participants with suspected p-PD and in none of the controls.

### 3.2. Non-Motor Features Distinguishing Suspected p-PD and Controls

**Figure 2:**
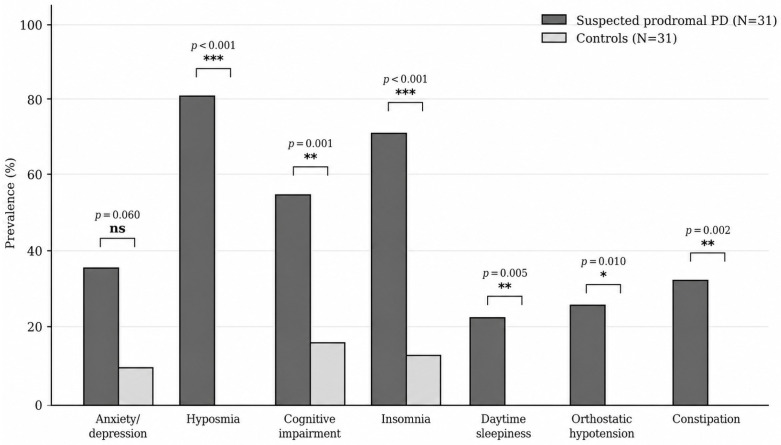
Prevalence (%) of non-motor features in participants in the suspected p-PD group and matched controls. Hyposmia and OH were predefined enrichment markers used for participant classification and are presented for descriptive purposes only. Between-group comparisons were performed using Fisher’s exact test. Significance levels: ns, not significant; * *p* < 0.05; ** *p* < 0.01; *** *p* < 0.001.

Individuals with suspected p-PD exhibited a broad, non-motor phenotype that clearly differentiated them from controls. Beyond the higher rates of insomnia (71.0% vs. 12.9%; *p* < 0.001) and EDS (22.6% vs. 0%; *p* = 0.005), cognitive impairment was more frequent (MoCA below threshold in 54.8% vs. 16.1%; *p* = 0.001). Mood symptom severity was also higher in the suspected p-PD group, reflected by higher HADS anxiety/depression scores (*p* < 0.001). Although a greater proportion of participants met criteria for at least mild depression or anxiety (35% vs. 10%), this difference did not reach statistical significance (*p* = 0.06). Because hyposmia and OH were incorporated as predefined enrichment markers for the suspected p-PD group, between-group differences in these variables were expected by design and are presented descriptively rather than interpreted as independent findings. As expected from the predefined enrichment strategy, OH was present in 25.8% (8/31) of participants with suspected p-PD and in none of the controls (0/31; Fisher’s exact test, *p* = 0.01). In contrast, constipation was significantly more common in the suspected p-PD group (32.3% vs. 0%; *p* = 0.002). Similarly, olfactory dysfunction was observed almost exclusively in the suspected p-PD group (hyposmia 80.6%, anosmia 3.2%), consistent with its role as a predefined enrichment marker, whereas all controls were normosmic (*p* < 0.001). Taken together, these observations were reflected in a significantly higher global non-motor burden (NMSQ) in the suspected p-PD group (severity distribution: 25.8% mild, 45.2% moderate, 25.8% severe, 3.2% very severe) than in controls (67.7% mild, 32.3% moderate; |Z| = 2.743, *p* = 0.006), as illustrated in Figure 2.

### 3.3. Sleep and Non-Motor Correlations in Participants with Suspected p-PD

Within the suspected p-PD group, Spearman analyses suggested associations between sleep and non-motor features. Higher global non-motor burden (NMSQ) correlated with worse mood; greater insomnia severity; higher restless leg severity; more daytime sleepiness; and olfactory, cognitive, Berlin BQ risk, and bowel habit measures. Olfactory dysfunction (SS-12) was associated with multiple domains: insomnia, mood, daytime sleepiness, cognitive impairment, and constipation. These findings suggest associations between hyposmia and poorer sleep, mood, cognition, and autonomic gut function. Detailed correlations of these measures are shown in Table 3.

## 4. Discussion

Our findings suggest that several NMS tend to co-occur in individuals with suspected p-PD. The cohort was intentionally enriched using RBD symptoms together with hyposmia and/or OH, as these markers have among the highest positive likelihood ratios within the updated MDS Research Criteria for p-PD [3]. This enrichment strategy was chosen to increase the likelihood of identifying individuals with a higher estimated probability of p-PD rather than to evaluate the discriminatory value of these markers themselves. Consequently, the observed between-group differences in hyposmia and OH should be regarded as expected consequences of the study design rather than independent findings. The more informative observations concern the increased burden of insomnia, EDS, cognitive impairment, mood symptoms, and constipation within this enriched prodromal phenotype, reinforcing the concept of PD as a multisystem disorder with a long non-motor prodromal phase. Recent evidence further supports the concept that NMS do not occur in isolation. Mota Telles et al. demonstrated significant associations between constipation, depressive symptoms, RBD, and disease severity, suggesting that these manifestations may reflect interconnected neurodegenerative pathways [16]. These features reinforce the view of PD as a multisystem disorder with a long prodrome [10,11]. In addition to the well-established specificity of RBD for future α-synucleinopathy [17], our data further indicate that EDS and insomnia are common in suspected p-PD. Recently, Ophey et al. emphasized that isolated RBD represents one of the strongest prodromal manifestations of α-synucleinopathy, while also highlighting the importance of integrating additional prodromal markers when applying current MDS criteria [18]. This supports our strategy of enriching the cohort with RBD symptoms together with hyposmia and/or OH to identify individuals with a higher probability of p-PD. Previous population-based studies have consistently demonstrated that EDS may precede the diagnosis of PD. Abbott et al. reported an increased risk of future PD among individuals with EDS, while Lysen et al. observed similar associations in a large community cohort [19,20]. Our findings support these observations by demonstrating significantly higher ESS scores and a greater prevalence of pathological daytime sleepiness in the suspected p-PD group. Despite evidence that poorer sleep quality increases the estimated probability of p-PD according to the MDS framework [3], insomnia has received less attention in previous studies [3,8]. Our cohort demonstrates its relevance to the broader burden of NMS. Consistent with our findings, cohorts defined by RBD and by hyposmia show an expanded cognitive impairment, most notably affecting executive and visuo-constructive functions [21]. Similarly, we observed a substantially higher frequency of cognitive impairment in the suspected p-PD group, supporting the growing evidence that cognitive dysfunction may emerge early in the suspected prodromal phase and coexist with other non-motor manifestations. OSA did not differ between groups—consistent with reports showing a similar or even lower prevalence of OSA in PD than in controls, taking into account key confounding factors (e.g., body mass index) [22,23,24]. In addition, the prodromal specificity of RLS remains unclear, its pathogenesis and proposed overlap with PD using iron dysregulation models are debated, and longitudinal evidence is conflicting [25,26,27]. As expected from the enrichment strategy, OH was present in 25.8% in our study, which is higher than some reports for the prodromal phase. In a Dutch population-based study, OH was not associated with increased PD risk in the general population, although it was more frequent among individuals with established PD. These findings raise questions about the specificity of OH as a prodromal marker [28,29,30]. The observed association between olfactory dysfunction and both cognitive impairment and depressive symptoms is consistent with the prior literature [31,32]. Within the suspected p-PD group, exploratory correlation analyses showed that olfactory dysfunction was associated with sleep disturbances and bowel dysfunction, suggesting that hyposmia may represent a marker of a broader multisystem prodromal phenotype rather than an isolated sensory deficit. Prospective and cross-sectional studies link smell loss with worse cognition and greater non-motor burden in PD [33,34,35]. More broadly, reviews and meta-analytic work indicate that hyposmia is an early, sensitive, but nonspecific prodromal feature associated with subsequent PD, reinforcing its utility in risk stratification [36,37]. The observed interrelationships, particularly the associations between hyposmia and worse sleep, mood, cognition, and bowel function, are consistent with models proposing that early involvement of brainstem, olfactory, and autonomic networks contribute to a broad prodromal phenotype and may improve risk-enrichment strategies for early intervention trials [11,38,39,40]. In conclusion, these findings support RBD-based case-finding combined with systematic assessment of insomnia, EDS, olfaction, autonomic symptoms, mood, and cognition to improve suspected p-PD phenotyping. Future longitudinal studies are required to determine whether this approach improves risk stratification and facilitates recruitment for disease-modifying trials.

### Limitations

First, RBD was identified using the validated RBD-I rather than v-PSG. Therefore, participants should be considered to have suspected rather than confirmed RBD. Second, no imaging biomarkers (e.g., DAT-SPECT), as well as biological (e.g., α-synuclein seeding assays) and genetic biomarkers were available to validate prodromal status. Third, the sample size was modest and no formal a priori sample size calculation was performed because the present study represents a secondary analysis of an existing population-based screening cohort. Fourth, only the overall HADS classification was available for analysis, precluding separate evaluation of anxiety and depression symptoms. Future studies should analyze HADS-A and HADS-D subscales independently to better characterize affective manifestations in suspected p-PD. Fifth, we did not perform multivariable (logistic) modeling because the number of events was small, several predictors exhibited complete separation (0% in controls), and hyposmia/OH were part of the case definition (incorporation bias), making any fitted model unstable and potentially misleading. Sixth, selection bias wass possible: of 159 RBD-I screen-positive invitees, only 62 participated, and those who opted in may have had a higher NMS burden. Seventh, multiple pairwise correlations were examined without formal correction for multiple testing. Consequently, some statistically significant associations may represent chance findings and should be interpreted as exploratory until replicated in larger independent cohorts. These limitations do not invalidate the main results but temper the strength of the conclusions. Future work should incorporate v-PSG confirmation, biomarker enrichment (e.g., DAT imaging, α-synuclein seeding assays, cardiac MIBG), and longitudinal follow-up to estimate phenoconversion and validate whether aggregated NMS burden, including insomnia/EDS, improves prognostic performance over single markers.

## 5. Conclusions

In an RBD-screened cohort enriched by hyposmia and/or OH, individuals with suspected p-PD exhibited a substantially greater burden of NMS than matched controls. The most informative findings were the higher prevalence of insomnia, EDS, cognitive impairment, mood symptoms, and constipation, supporting the concept of a broad suspected prodromal phenotype. Within the suspected p-PD group, these non-motor domains were associated with one another, suggesting that these manifestations tend to co-occur rather than occur in isolation. These findings support a comprehensive assessment of sleep, cognition, mood, autonomic symptoms, and olfactory function, which may improve early risk stratification beyond single-marker approaches. Future longitudinal studies are needed to determine whether these combined non-motor markers predict conversion to clinically manifest PD.

## Figures and Tables

**Table 1:** Evaluation thresholds of sleep questionnaires. Abbreviations: pts—points, RLS—Restless Legs Syndrome.

Sleep Questionnaires	Minimum and Maximum Scores, Pathological Values
Insomnia Severity Index	0 ÷ 28 pts.
	0–7 pts.—none
	8–14 pts.—subthreshold
	15–21 pts.—moderate
	22–28 pts.—severe
Berlin Sleep Apnea Questionnaire	Questions divided into three categories:
	≥2 positive categories—high risk
	≤1 positive category—low risk
Restless Legs Syndrome (RLS) Rating Scale	0 ÷ 40 pts.
	0 pts.—no RLS
	1–10 pts.—mild RLS
	11–20 pts.—moderate RLS
	21–30 pts.—severe RLS
	31–40 pts.—very severe RLS
Epworth Sleepiness Scale	0 ÷ 24 pts.
	<10 pts.—normal
	≥10 pts.—pathological sleepiness

**Table 2:** Demographic and clinical characteristics of the suspected p-PD group and controls (*n* = 31 per group). Abbreviations: *n*—number of participants; $\bar{x}$ ± SD—mean ± standard deviation; p-PD—prodromal Parkinson’s disease.

Variable	Descriptive Statistics		
	Suspected p-PD *n* = 31	Controls *n* = 31	*p* Value
Age, $y,\;\bar{x}$ ± SD	46.48 ± 14.44	46.42 ± 14.44	1.000
Sex			1.000
Men, *n* (%)	15 (48.4)	15 (48.4)	
Women, *n* (%)	16 (51.6)	16 (51.6)	
Education level			0.493
Primary, *n* (%)	1 (3.2)	0 (0)	
Secondary or vocational, *n* (%)	15 (48.4)	18 (58.1)	
Higher education, *n* (%)	15 (48.4)	13 (41.9)	
Marital status			0.603
Married, *n* (%)	17 (54.8)	14 (45.2)	
Single, *n* (%)	5 (16.1)	4 (12.9)	
Divorced, *n* (%)	5 (16.1)	7 (22.6)	
Widowed, *n* (%)	1 (3.2)	0 (0)	
Cohabiting (unmarried), *n* (%)	3 (9.7)	6 (19.4)	
Occupation (type of work)			0.874
Industry/construction, *n* (%)	4 (12.9)	4 (12.9)	
Office/professional work, *n* (%)	6 (19.4)	8 (25.8)	
Healthcare sector, *n* (%)	2 (6.5)	1 (3.2)	
Student, *n* (%)	1 (3.2)	0 (0)	
Homemaker, *n* (%)	1 (3.2)	0 (0)	
Not working (retired or disabled), *n* (%)	4 (12.9)	4 (12.9)	
Unemployed, *n* (%)	2 (6.5)	1 (3.2)	
Self-employed, *n* (%)	3 (9.7)	5 (16.1)	
Caring for a child (parental leave), *n* (%)	0 (0)	1 (3.2)	
Service sector *n* (%)	8 (25.8)	7 (22.6)	
Work with metals/contaminated environment			0.875
No, *n* (%)	21 (67.7)	21 (67.7)	
Yes, current, *n* (%)	2 (6.5)	3 (9.7)	
Yes, previously, *n* (%)	8 (25.8)	7 (22.6)	
Childhood residence			0.554
Urban, *n* (%)	29 (93.5)	30 (96.8)	
Rural, *n* (%)	2 (6.5)	1 (3.2)	
Childhood drinking water source			0.746
Municipal tap water, *n* (%)	20 (64.5)	21 (67.7)	
Well, *n* (%)	10 (32.3)	8 (25.8)	
Public hydrant, *n* (%)	1 (3.2)	2 (6.5)	
Dominant hand			0.374
Left, *n* (%)	9 (29.0)	6 (19.4)	
Right, *n* (%)	22 (71.0)	25 (80.6)	
Physical activity			0.439
4–7 times per week, *n* (%)	5 (16.1)	4 (12.9)	
1–3 times per week, *n* (%)	13 (41.9)	9 (29.0)	
Rarely or never, *n* (%)	13 (41.9)	18 (58.1)	
Family history of PD			0.520
Present, *n* (%)	7 (22.6)	5 (16.1)	
Absent, *n* (%)	24 (77.4)	26 (83.9)	

**Table 3:** Spearman correlation matrix of sleep measures and non-motor features in the suspected p-PD group (*n* = 31). Cells report Spearman’s correlation coefficient (ρ) (top) and two-tailed *p*-value (bottom). Bold values indicate *p* < 0.05. Correlation analyses were exploratory, and no correction for multiple comparisons was applied. Abbreviations: NMSQ—Non-Motor Symptoms Questionnaire (total), ESS—Epworth Sleepiness Scale, RLSRS—Restless Legs Syndrome Rating Scale, BQ—Berlin Questionnaire (obstructive sleep apnea risk), MoCA—Montreal Cognitive Assessment, SS-12—12-item smell identification test (olfaction), BSFS—Bristol Stool Form Scale, HADS—Hospital Anxiety and Depression Scale (total), ISI—Insomnia Severity Index, OH—Orthostatic hypotension.

Measures	NMSQ, ρ, *p*	ESS, ρ, *p*	RLSRS, ρ, *p*	BQ, ρ, *p*	MoCA, ρ, *p*	SS-12, ρ, *p*	BSFS, ρ, *p*	HADS, ρ, *p*	ISI, ρ, *p*	OH, ρ, *p*
NMSQ, ρ, *p*	1.000	**0.317** **0.012**	**0.426** **0.001**	**0.265** **0.037**	**0.323** **0.010**	**0.395** **0.002**	−**0.290** **0.022**	**0.578** **0.001**	**0.515** **0.001**	−**0.298** **0.019**
ESS, ρ, *p*	**0.317** **0.012**	1.000	−0.147 0.256	−0.164 0.201	−0.055 0.673	**0.305** **0.016**	−0.118 0.361	**0.289** **0.023**	0.089 0.494	−0.167 0.195
RLSRS, ρ, *p*	**0.426** **0.001**	−0.147 0.256	1.000	0.224 0.080	**0.297** **0.019**	0.062 0.633	−0.197 0.124	0.244 0.056	**0.299** **0.018**	0.029 0.826
BQ, ρ, *p*	**0.265** **0.037**	−0.164 0.201	0.224 0.080	1.000	**0.385** **0.002**	0.178 0.165	−0.204 0.113	0.079 0.544	**0.417** **0.001**	−0.180 0.161
MoCA, ρ, *p*	**0.323** **0.010**	−0.055 0.673	**0.297** **0.019**	**0.385** **0.002**	1.000	**0.410** **0.001**	−**0.462** **0.001**	0.170 0.185	**0.418** **0.001**	−0.110 0.395
SS-12, ρ, *p*	**0.395** **0.002**	**0.305** **0.016**	0.062 0.633	0.178 0.165	**0.410** **0.001**	1.000	−**0.410** **0.001**	**0.396** **0.001**	**0.504** **0.001**	−0.097 0.453
BSFS, ρ, *p*	−**0.290** **0.022**	−0.118 0.361	−0.197 0.124	−0.204 0.113	−**0.462** **0.001**	−**0.410** **0.001**	1.000	−0.168 0.193	−**0.269** **0.035**	0.037 0.774
HADS, ρ, *p*	**0.578** **0.001**	**0.289** **0.023**	0.244 0.056	0.079 0.544	0.170 0.185	**0.396** **0.001**	−0.168 0.193	1.000	**0.370** **0.003**	−**0.285** **0.025**
ISI, ρ, *p*	**0.515** **0.001**	0.089 0.494	**0.299** **0.018**	**0.417** **0.001**	**0.418** **0.001**	**0.504** **0.001**	−**0.269** **0.035**	**0.370** **0.003**	1.000	−0.087 0.503
OH, ρ, *p*	−**0.298** **0.019**	−0.167 0.195	0.029 0.826	−0.180 0.161	−0.110 0.395	−0.097 0.453	0.037 0.774	−**0.285** **0.025**	−0.087 0.503	1.000

## Data Availability

The original contributions presented in this study are included in the article. Further inquiries can be directed to the corresponding author.

## Abbreviations

The following abbreviations are used in this manuscript:

AI	Artificial Intelligence
BP	Blood Pressure
BQ	Berlin Questionnaire
BSFS	Bristol Stool Form Scale
DBP	Diastolic Blood Pressure
DAT-SPECT	Dopamine Transporter Single-Photon Emission Computed Tomography
EDS	Excessive Daytime Sleepiness
ESS	Epworth Sleepiness Scale
HADS	Hospital Anxiety and Depression Scale
HR	Heart Rate
IBM	International Business Machines
ISI	Insomnia Severity Index
IRLS	Restless Legs Syndrome Rating Scale
LUHS	Lithuanian University of Health Sciences
MDS	Movement Disorder Society
MIBG	Meta-Iodobenzylguanidine
MoCA	Montreal Cognitive Assessment
NMS	Non-Motor Symptoms
NMSQ	Non-Motor Symptoms Questionnaire
OH	Orthostatic Hypotension
OSA	Obstructive Sleep Apnea
PD	Parkinson’s Disease
PLM	Periodic Limb Movement
p-PD	Prodromal Parkinson’s Disease
PSG	Polysomnography
RBD	REM Sleep Behavior Disorder
RBD-I	REM Sleep Behavior Disorder Inventory
RLS	Restless Legs Syndrome
SBP	Systolic Blood Pressure
SD	Standard Deviation
SS-12	12-item Smell Identification Test
v-PSG	Video-Polysomnography
